# Supplementary material for: A Comparison between Perceptions of Psychiatric Outpatients and Psychiatrists Regarding Benzodiazepine Use and Decision Making for Its Discontinuation: A Cross-Sectional Survey in Japan
Source: Int J Environ Res Public Health. 2023 Apr 3;20(7):5373. doi: 10.3390/ijerph20075373 (PMC10094391; doi:10.3390/ijerph20075373)
Supplement: Supplementary file 1 [file ijerph-20-05373-s001.zip › ijerph-2218178-supplementary.pdf]

For patients

- Age: 20’, 30’, 40’, 50’, 60’, 70’, over80

- Sex: Female, Male, Other

- Service used: Clinic, Psychiatric hospital, General hospital, University hospital, Other.

- Diagnosis*: Schizophrenia, Bipolar disorder, Major depressive disorder, Anxiety disorder, Developmental disorder, Insomnia, Other, Unknown

- Experience of taking hypnotic/anxiolytic: Hypnotic only, Anxiolytic only, Both

- Duration of taking hypnotic: <1 month, 1-3 months, 3-6 months, 6-12 months, ≥ 12 months.

- Numbers taking hypnotic: 1, 2, 3, ≥ 4

- Duration of taking anxiolytic: <1 month, 1-3 months, 3-6 months, 6-12 months, ≥ 12 months.

- Numbers taking anxiolytic: 1, 2, 3, ≥ 4

- Desirable timing of hypnotic/anxiolytic reduction after symptom improvement:
  -Immediately
  -Within 3 months
  -Within 6 months
  -Within 12 months
  -After 12 months passed
  -No need to reduce if there are no side effects
  -Other
  -Unknown

- Permissible situation of hypnotic/anxiolytic continuation**:
  -When I am still suffering from symptoms of insomnia or anxiety
  -When my social functioning is disrupted
  -When I still have physical or mental problem, which led to start to take the medication
  -When I want to continue the medication
  -When I am not suffering from side effects of the medication
  -When my quality of life is worsened
  -When the number of medication I am (is) taking is low
  -Other
  -Unknown

For patients

- Decision making regarding continuing/tapering hypnotic/anxiolytic use
  -The decision is (was) made, considering the patient's opinion
  -The outpatient and psychiatrist make (made) the decision together, on an equal basis
  -The decision is (was) made, considering the psychiatrist's opinion

- Difficulties cited while attempting to reduce hypnotic/anxiolytic in the past*:
  -I could not reduce the medication because the patient was unwilling to reduce it
  -I did not know how to reduce the medication due to insufficient related information (provided by psychiatrist)
  -I did not know when I should reduce the medication due to insufficient related information (provided by psychiatrist)
  -I did not know the situation or condition, where the medication can be reduced due to insufficient related information (provided by psychiatrist)
  -I could not reduce the medication because of worsening symptoms
  -I could not reduce the medication because withdrawal symptoms (e.g. tremors, palpitations, anxiety) appeared when I tried to reduce
  -I never had any particular problem regarding the medication reduction
  -I never reduced the mediation
  -Other

*multiple answers
**multiple answers up to three

For psychiatrists

- Age: 20', 30', 40', 50', 60', 70', over80

- Sex: Female, Male, Other

- Affiliation: Clinic, Psychiatric hospital, General hospital, University hospital, Other.

- Desirable timing of hypnotic/anxiolytic reduction after symptom improvement:
  -Immediately
  -Within 3 months
  -Within 6 months
  -Within 12 months
  -After 12 months passed
  -No need to reduce if there are no side effects
  -Other
  -Unknown

- Permissible situation of hypnotic/anxiolytic continuation**:
  -When the patient is still suffering from symptoms of insomnia or anxiety
  -When the patient's social functioning is disrupted
  -When the patient still has physical or mental problem, which led to start to take the medication
  -When the patient wants to continue the medication
  -When the patient is not suffering from side effects of the medication
  -When the patient's quality of life is worsened
  -When the number of medication the patient is taking is low
  -Other
  -Unknown

- Decision making regarding continuing/tapering hypnotic/anxiolytic use
  -The decision is (was) made, considering the patient's opinion
  -The outpatient and psychiatrist make (made) the decision together, on an equal basis
  -The decision is (was) made, considering the psychiatrist's opinion

- Difficulties cited while attempting to reduce hypnotic/anxiolytic in the past*:
  -I could not reduce the medication because the patient was unwilling to reduce it
  -I could not reduce the medication because the patient was unwilling to reduce it
  -I did not know how to reduce the medication due to insufficient related information (provided by psychiatrist)
  -I did not know when I should reduce the medication due to insufficient related information (provided by psychiatrist)

For psychiatrists

- I did not know the situation or condition, where the medication can be reduced due to insufficient related information (provided by psychiatrist)
- I could not reduce the medication because of worsening symptoms
- I could not reduce the medication because withdrawal symptoms (e.g., tremors, palpitations, anxiety) appeared when I tried to reduce
- I never had any particular problem regarding the medication reduction
- I never reduced the mediation
- Other

*multiple answers

**multiple answers up to three
